# Exposure to OPFRs Is Associated with Obesity and Dysregulated Serum Lipid Profiles: Data from 2017–2018 NHANES

**DOI:** 10.3390/metabo14020124

**Published:** 2024-02-13

**Authors:** He Li, Fenglin Li, Chaoyi Zhou, Jifan Bu, Hao Yang, Liangchen Zhong, Weilong Xing, Liangzhong Li

**Affiliations:** 1 School of Civil Engineering, Southeast University, Nanjing 210096, China; 101010868@seu.edu.cn (H.L.); 220215093@seu.edu.cn (F.L.); 220224670@seu.edu.cn (J.B.); 220221447@seu.edu.cn (H.Y.); 220215097@seu.edu.cn (L.Z.); 2 Laboratory of Pesticide Environmental Assessment and Pollution Control, Nanjing Institute of Environmental Sciences, Ministry of Ecology and Environment (MEE), Nanjing 210042, China; 221211901129@stu.just.edu.cn; 3 School of Environmental and Chemical Engineering, Jiangsu University of Science and Technology, Zhenjiang 212003, China; 4 CAS Key Laboratory of Renewable Energy, Guangdong Provincial Key Laboratory of New and Renewable Energy Research and Development, Guangzhou Institute of Energy Conversion, Chinese Academy of Sciences, Guangzhou 510640, China

**Keywords:** flame retardant, obesity, BMI, serum lipid, NHANES

## Abstract

Widespread exposure to organophosphorus flame retardants (OPFRs) has been observed in the general population. Emerging studies have revealed OPFRs possess endocrine-disturbing properties. The present study aims to assess the association between urinary metabolites of OPFRs, BMI, and serum lipid profiles. Data from the National Health and Nutrition Examination Survey (NHANES) 2017–2018 were obtained, with 1334 adults enrolled in the current study. Urinary concentrations of bis (1-chloro-2-propyl) phosphate (BCIPP), bis(2-chloroethyl) phosphate (BCEP), bis(1,3-dichloro-2-propyl) phosphate (BDCPP), dibutyl phosphate (DBUP), and diphenyl phosphate (DPHP) were quantified to assess OPFR exposure. Covariate-adjusted linear and logistic regression models were conducted to explore the associations between log_2_-transformed concentrations of OPFR metabolites, BMI, obesity, and serum lipid profiles. Stratified analyses were performed to assess the heterogeneity of associations by age, gender, race, etc. Positive associations were found between OPFR exposure and the risk of obesity. The multivariate linear analysis indicated that a one-unit increase in log_2_-transformed urinary concentrations of BCEP and BDCPP was associated with 0.27 (95% CI: 0.02–0.52, *p* = 0.0338) and 0.56 (95% CI: 0.25–0.87, *p* = 0.0004) higher BMI value, respectively. One log_2_-unit increase in urinary BCEP and BDCPP concentrations was associated with 1.1-fold (95% CI: 1.02–1.18, *p* = 0.0096) and 1.19-fold (95% CI: 1.09–1.30, *p* = 0.0001) risk for developing obesity. Furthermore, the non-linear relationship between exposure to OPFRs and obesity was identified. Additionally, multivariable linear regression showed that urinary DPHP concentrations were inversely correlated with serum triglyceride (TG) levels (β = −7.41, 95% CI: −12.13 to −2.68, *p* = 0.0022). However, no other OPFR metabolites were found to be significantly statistically associated with serum lipid levels after adjusting for potential confounders. In conclusion, environmental exposure to OPFRs might contribute to obesity and dysregulated TG concentrations in adults. Future prospective research is warranted to confirm the causal relationship between metabolites of OPFRs and obesity.

## 1. Introduction

Flame retardants are a class of chemical additives designed to reduce the flammability of products [1]. Polybrominated diphenyl ethers (PBDEs) have been phased out since 2004 due to their toxicity, environmental persistence, and bio-accumulative properties [2]. Organophosphorus flame retardants (OPFRs) have been introduced to the market and acted as substitutes for PBDE. Nowadays, OPFRs find wide applications in plastics, furniture, textiles, glues, vehicles, electrical equipment, and electronic devices [3]. Monitoring studies revealed OPFR exposure was widespread among the general population. They can be ubiquitously detected in water [4], air [5], and sediment [6]. Regrettably, emerging evidence suggested that exposure to OPFRs has adverse effects on human health [7], including endocrine disturbance [8,9,10], nephrotoxicity [11], neurotoxicity [12], and carcinogenicity [13]. The widespread exposure to OPFRs and their associated toxicity properties raise concerns about whether OPFRs are a safer replacement for PBDE.

Obesity has become a global public health concern, with a dramatically increasing prevalence rate over the past two decades [14]. According to the World Health Organization (WHO), more than 650 million adults globally are affected by obesity. This condition is linked to the development of various cancers, cardiovascular diseases, and diabetes [15], ranking as the 5th most common cause of death [16]. Body mass index (BMI) is a widely used and simple index for evaluating obesity [17] and has a cut-off value of 30 kg/m^2^ according to the WHO criteria [18]. Environmental factors are increasingly recognized as major contributors to the obesity epidemic as excessive energy intake and insufficient physical activity alone could not fully explain the sharp increase in obesity prevalence [19]. Notably, exposure to endocrine-disrupting chemicals, such as di-2-ethylhexyl phthalate (DEHP), has been associated with weight gain in adulthood [20]. Obesity is closely associated with dyslipidemia in adults, and the prevalence of dyslipidemia is on the rise [21]. Intriguingly, accumulating evidence suggests that exposure to endocrine-disrupting chemicals could disturb lipid homeostasis [22]. Recent in vitro studies have confirmed that exposure to both classic and novel brominated flame retardants may promote triglyceride accumulation in preadipocytes and hepatocytes [23,24]. However, studies evaluating the relationship between OPFR metabolites, disturbance in lipid metabolism, and obesity among the general population are limited. This study aims to evaluate whether exposure to OPFR metabolites contributes to obesity among 1334 participants undergoing the National Health and Nutrition Examination Survey (NHANES). NHANES recently developed a method [25] to measure the metabolites of OPFRs, which serve as exposure biomarkers: bis (1-chloro-2-propyl) phosphate (BCIPP), bis(2-chloroethyl) phosphate (BCEP), bis(1,3-dichloro-2-propyl) phosphate (BDCPP), dibutyl phosphate (DBUP), diphenyl phosphate (DPHP), and 2,3,4,5-tetrabromobenzoic acid (TBBA). Therefore, NHANES 2017–2018 cycle data were obtained in this study, as OPFR metabolites were measured using this novel method. Additionally, the effect of OPFR metabolites on obesity was evaluated, stratified by age, sex, race, etc. Furthermore, the associations between urinary concentrations of OPFR metabolites and blood lipid profiles were explored.

## 2. Methods

### 2.1. Study Population

**Figure 1:**
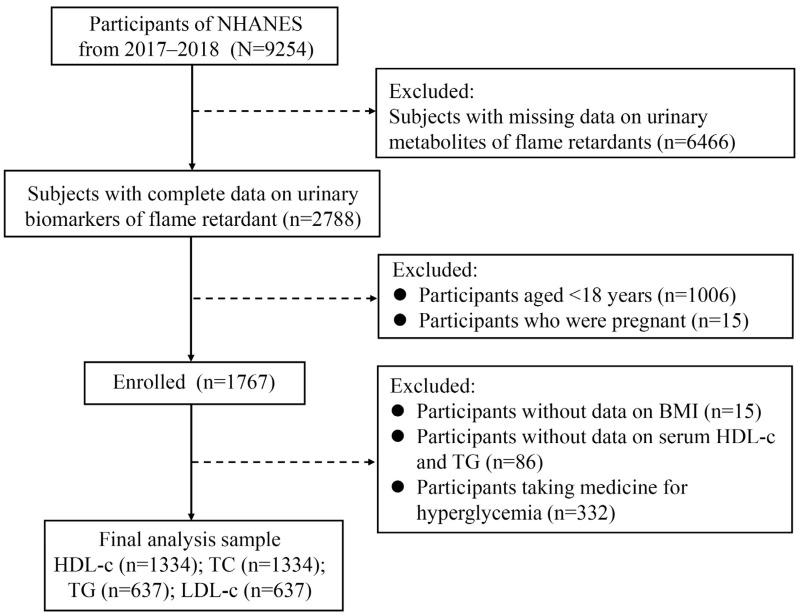
Flow diagram of the study population. NHANES, National Health and Nutrition Examination Survey; HDL-c: high-density lipoprotein cholesterol; TC: total cholesterol; TG: triglyceride; LDL-c: low-density lipoprotein cholesterol.

NHANES is a cross-sectional nationally representative survey focusing on the health and nutritional status of adults and children in the general non-institutionalized population of the United States, utilizing a complex, multistage probability sampling design [26]. The survey collects demographic characteristics, anthropometric data, dietary supplements, questionnaire information, and laboratory parameters. The flow chart of the study population is illustrated in Figure 1. The current study included adults aged over 18 with complete data for analysis, resulting in 9254 participants from NHANES 2017–2018. Participants with absent urinary concentrations of OPFRs (*n* = 6466), those aged less than 18, and pregnant females were excluded. Among the remaining 1767 participants, only those with complete data on BMI, HDL-c, triglyceride (TG), and not taking medicine for hyperglycemia were ultimately included in the study. All procedures were approved by the National Center for Health Statistics Research Ethics Committee, and written informed consent was obtained from all participants at recruitment.

### 2.2. Detection of Urinary Concentrations of OPFR Biomarkers

Spot urine specimens of 0.2 mL were collected, processed, and stored in −30 °C conditions until shipment to the CDC National Center for Environmental Health for analysis. Six types of urinary OPFR metabolites, namely BCIPP, BCEP, BDCPP, DBUP, DPHP, and TBBA, were quantified as exposure biomarkers of OPFRs. The analytical method involves enzymatic hydrolysis of urinary conjugates of target analytes, automated off-line solid phase extraction, and isotope dilution high-performance liquid chromatography–tandem mass spectrometry detection [27]. NHANES quality assurance and quality control (QA/QC) protocols adhere to the 1988 Clinical Laboratory Improvement Amendments mandates. A detailed description of the sample preparation and instrument parameters can be found at https://wwwn.cdc.gov/nchs/data/nhanes/2017-2018/labmethods/FR-J-MET-508.pdf (accessed on 2 December 2023). The lower limits of detection (LLODs) for urinary BCIPP, BDCPP, DBUP, and DPHP were 0.1 ng/mL, while the LLOD for urinary TBBA was 0.05 ng/mL. TBBA was excluded in subsequent analyses due to 93.78% of samples being below the LLOD. Urinary creatinine concentrations were measured to account for dilution-dependent sample variation in biomarker concentrations.

### 2.3. The Definition of Outcomes

BMI values were extracted from body measure data from NHANES. The primary outcomes of the present study were BMI value and the occurrence of obesity. Obesity was defined as BMI ≥ 30 kg/m^2^ [28]. The secondary endpoints were the serum lipid profiles, which included TG, total cholesterol (TC), high-density lipoprotein cholesterol (HDL-c), and low-density lipoprotein cholesterol (LDL-c).

### 2.4. Covariates

Covariates included age, sex, race, smoking status, drinking status, physical activity, education level, marital status, family poverty income ratio (PIR), and urinary creatinine, based on previous literature [29,30]. Additionally, independent risk factors identified from univariate linear analysis for BMI, TG, TC, HDL-c, and LDL-c were screened as the potential covariates for the present research. The participants belonged to five ethnic groups: Mexican American, other Hispanic, non-Hispanic White, non-Hispanic Black, and other race (including multiracial). Smoking status, drinking status, and physical activity were obtained from self-reported questionnaire data. Smoking status was categorized as current smoker (smoked at least 100 cigarettes and is smoking currently), former smoker (smoked at least 100 cigarettes and has quit smoking currently), and non-smoker (smoked less than 100 cigarettes in their entire life). Drinking status was dichotomized into drinker (drinks at least 12 alcoholic drinks per year) and non-drinker (drinks fewer than 12 alcoholic drinks per year). Physical activity was categorized as none, moderate activity, and vigorous activity [29]. Education level was grouped as less than 9th grade, 9th–11th grade, high school graduate/GED or equivalent, some college or AA degree, and college graduate or above. Marital status was classified into married/living with a partner, widowed/divorced/separated, and never married. Family PIR was divided into 4 groups: <1, 1–1.99, 2–3.99, and ≥4 [31].

### 2.5. Statistical Analysis

Normally distributed variables were presented as mean ± standard deviation, while non-normally distributed data were expressed as the median and interquartile range (Q1–Q3). Specially, urinary concentrations of OPFR metabolites were presented as geometric means (GMs) and 95% confidence interval (CI). Categorical variables were described using numbers and percentages. Student *t*-test, Mann–Whitney test, and chi-square test were employed to compare differences in continuous and categorical variables. Log_2_-transformed concentrations of metabolites were calculated to normalize their distribution and used in subsequent statistical analyses. Biomarker concentrations below the LOD were replaced with LOD divided by the square root of 2 [32]. Spearman’s correlation coefficients among these log_2_-transformed concentrations of OPFR metabolites were calculated. Given the complex sampling design in NHANES, survey weights were applied in statistical regression models [33]. Multivariate linear regression models were conducted to assess the associations between urinary metabolites of OPFRs, BMI, and serum lipid profiles after adjusting for confounders. Log_2_-transformed concentrations of urinary concentrations of metabolites of OPFRs were treated as independent variables. β and 95% CI were calculated. Multivariate logistic regression analyses were performed to evaluate the relationship between urinary concentrations of OPFR metabolites and obesity, with odds ratio (OR) and 95% CI calculated. Non-linear relationships between urinary OPFR metabolites and obesity were described using a generalized additive model (GAM) and smooth curve fitting. Subsequently, stratification analyses of associations between urinary BCEP/BDCPP concentrations and obesity were conducted. The stratification variables were chosen because previous reports revealed these factors were associated with obesity and serum lipid levels. All data analyses were conducted using R software (version 4.1.3) and GraphPad with NHANES-provided sampling weights. Significant levels were set at a two-tailed *p*-value of 0.05.

## 3. Results

### 3.1. Baseline Characteristics of Study Participants

Table 1 presents data from a total of 1334 individuals included in the present research. The mean age of the included subjects was 45.19 ± 17.59 years. Among them, 656 (49.18%) were male, and 678 (50.82%) were female. The majority of the subjects identified as non-Hispanic White and non-Hispanic Black. Over 60% reported current smoking, and 11% reported alcohol consumption. One in four individuals reported engaging in vigorous physical activity, while more than half of the participants had received a college education. Nearly three in five were either married or lived with a partner. The proportion of individuals with PIR less than 1 was 18.59% and one in four had hypertension. Additional details can be obtained from Table 1.

### 3.2. Overview of OPFR Metabolites in Urine Samples

**Figure 2:**
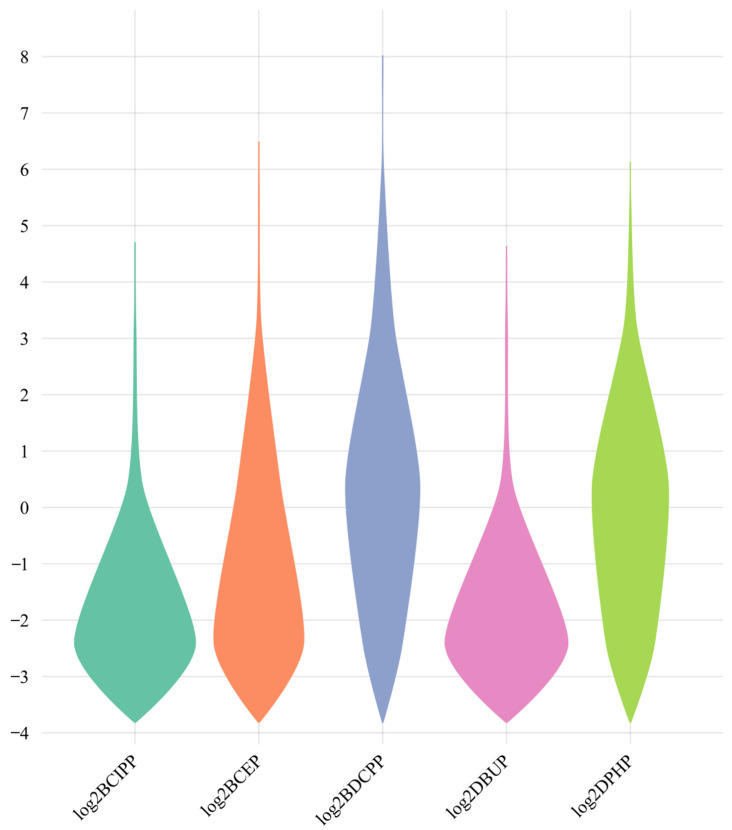
Distribution of urinary concentrations of OPFR metabolites among the whole population.

**Figure 3:**
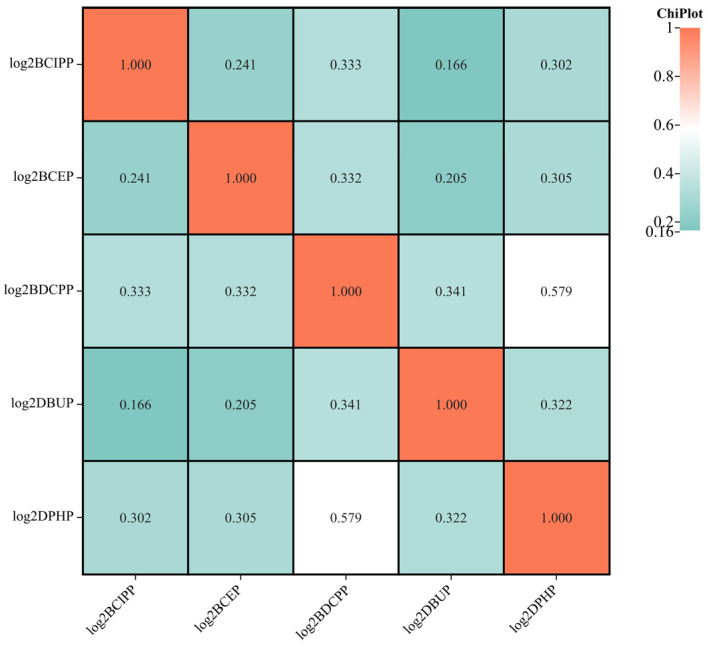
Spearman correlation among 5 kinds of OPFR metabolites.

An overview of OPFR metabolites in urine samples is presented in Table 2, including concentrations of urinary creatinine levels and log_2_-transformed urinary OPFR metabolites. The median concentration of creatinine in urine samples was 118 mg/dL. Figure 2 illustrates the distribution of log_2_-transformed urinary concentrations of OPFR metabolites across all participants. BCEP exhibited the highest GM (1.046 ng/mL) compared to other types of OPFR metabolites. It is noteworthy that only weak to moderate correlations were found among these OPFR biomarkers, with DPHP and BDCPP showing the highest correlation (r = 0.579, *p* < 0.01) as depicted in Figure 3.

### 3.3. Associations of Urinary OPFR Metabolites, BMI, and Obesity

The participants were equally divided into four quartiles based on the levels of five kinds of OPFR metabolites, and the distribution of BMI and serum lipid profiles across different groups is presented in Tables S1 and S2. Additionally, as shown in Table 3, a multiple linear regression model indicated that BCIPP, BCEP, BDCPP, and DBUP were all independently associated with increased BMI value after adjusting for age, sex, and race, with β coefficients of 0.32 (95% CI:0.03–0.62, *p* = 0.0323), 0.33 (95% CI: 0.09–0.57, *p* = 0.0068), 0.71 (95% CI: 0.46–0.96, *p* < 0.0001), and 0.35 (95% CI: 0.03–0.67, *p* = 0.00310), respectively. However, in the fully adjusted model, only BCEP and BDCPP were positively associated with BMI. A unit increase in log_2_BCEP was associated with 0.27 (95% CI: 0.02–0.52) higher BMI value (*p* = 0.0338), and a unit increase in log_2_BDCPP was associated with 0.56 (95% CI: 0.25–0.87) higher BMI value (*p* = 0.0004). Next, participants were categorized into obese (BMI ≥ 30 kg/m^2^) and non-obese (BMI < 30 kg/m^2^) groups. The effect of these metabolites on the risk of obesity was further explored using multivariable logistic regression, and the results are presented in Table 4. Specifically, a log_2_ unit increase in BCEP, BDCPP, DBUP, and DPHP was associated with 12% (95% CI: 1.05–1.20, *p* = 0.0005), 21% (95% CI: 1.09–1.30, *p* < 0.0001), 10% (95% CI: 1.01–1.20, *p* = 0.0273), and 9% (95% CI: 1.01–1.17, *p* = 0.0206) higher risk for obesity in the minimally adjusted model, respectively. BCEP and BDCPP were significantly associated with 1.10 (95% CI: 1.02–1.18, *p* = 0.0096) and 1.19 (95% CI: 1.09–1.30, *p* = 0.0001) odds ratios for obesity in the fully adjusted model. Additionally, the relationship between urinary BCEP/BDCPP concentrations and obesity is displayed in Figure S1 from a generalized additive model (GAM) and smooth curve fitting analysis. The results revealed the non-linear relationship between BCEP and obesity, as well as BDCPP and obesity.

### 3.4. Stratification Analyses of Associations of Urinary BCEP/BDCPP Concentrations and Obesity

**Figure 4:**
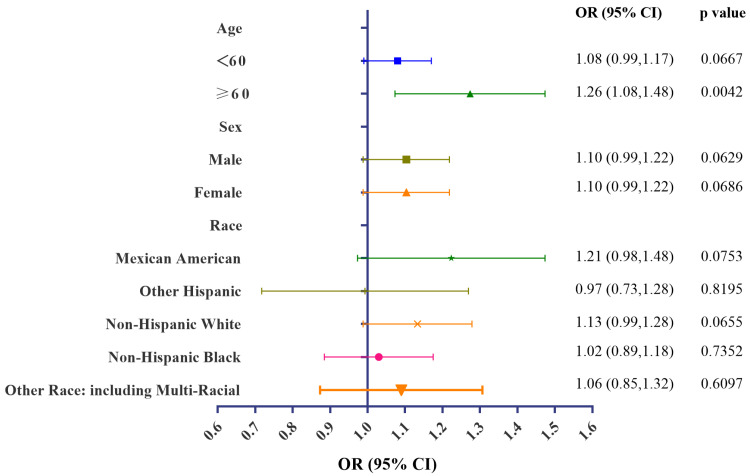
Stratification analyses of urinary BCEP concentrations and obesity.

**Figure 5:**
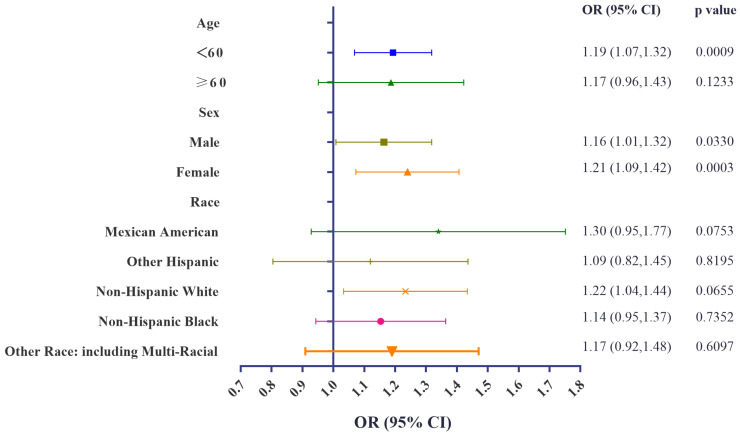
Stratification analyses of urinary BDCPP concentrations and obesity.

Subgroup analyses were conducted, stratified by age, sex, and race. Significant positive associations between urinary concentrations of BCEP and obesity remained in subjects aged over 60. Furthermore, urinary BDCPP concentrations remained associated with a higher risk for obesity among those aged less than 60, males, females, and non-Hispanic White participants. Detailed information can be obtained from Figure 4 and Figure 5. Collectively, these stratification variables influenced the relationship between OPFR exposure and obesity. Special attention needs to be given to subgroup participants with a higher risk of developing obesity under OPFR exposure. GAM and smooth curve fitting analysis were further performed to explore the associations between urinary BCEP/BDCPP and obesity. Non-linear relationships between urinary BCEP/BDCPP concentrations and obesity are presented in Figures S2–S10.

### 3.5. Association of Urinary Concentrations of OPFR Metabolites and Serum Lipid Profiles

The impact of OPFR exposure on serum lipid profiles, including TG, TC, HDL-c, and LDL-c was explored, and the findings are presented in Table 5. Interestingly, an inverse association was found between urinary BCEP concentrations and HDL-c (β = −0.52, 95% CI: −1.00 to −0.09, *p* = 0.0199), urinary BDCPP concentrations and HDL-c (β = −0.55, 95% CI: −1.04 to −0.07, *p* = 0.0250), urinary DPHP concentrations and TG (β = −5.67, 95% CI: −9.34 to −2.00, *p* = 0.0025), and urinary DPHP concentrations and HDL-c (β = −0.59, 95% CI: −1.08 to −0.08, *p* = 0.0226) in the model adjusted for age, sex, and race. In fully adjusted multivariable linear regression models, only DPHP was found to be inversely associated with serum TG levels. One-unit elevation of urinary log_2_DPHP was related to a 7.41 mg/dL lower level of TG.

## 4. Discussion

The present study investigated cross-sectional associations between BMI, obesity, serum lipid profiles, and urinary biomarker concentrations of five OPFR metabolites among general adults. These metabolites were frequently detected in urine samples, and both urinary BCEP and BDCPP were found to be positively associated with BMI value and the prevalence of obesity. In contrast, an inverse association was observed between urinary DPHP concentrations and serum TG levels. No significant relationships between other OPFRs, BMI, and serum lipid profiles were observed.

Previous epidemiological and laboratory studies on the associations between urinary OPFR metabolite concentrations and obesity are limited and inconsistent, providing an incomplete understanding of the effect of OPFR metabolites on obesity among the general adult population. Two studies in pregnant women indicated positive associations between select OPFR metabolites and BMI value, where obese pregnant women had higher concentrations of BCEP and BDCPP compared with those with normal weight [34,35]. In vivo evidence showed perinatal and postnatal exposure to Firemaster 550, a type of OPFR mixture, resulted in increased body weight in rats [36,37]. A recent study suggested that subjects with detectable BCPP had a higher risk of developing obesity in NHANES 2013–2014 compared to participants whose urinary BCPP concentrations were below the LLOD [38]. These findings supported our observed positive associations with higher BMI value and obesity for select urinary OPFR metabolites after adjusting for confounders. In contrast, two studies aiming to explore the relationship between certain OPFR metabolites and adiposity suggested that DBUP concentrations in urine samples were inversely associated with several adiposity markers (BMI, obesity, and waist circumference) among both children and adults [38,39]. The underlying mechanisms are not yet clarified, but it is suggested that tri-n-butyl phosphate (TNBP), acting as the parent compound of DBUP, tends to accumulate in adipose tissue, which might result in decreased urinary concentrations of DBUP [38].

An epidemiological study conducted across nine European countries detected concentrations of DPHP five times higher than other OPFR metabolites [40]. Similarly, our study observed relatively higher levels of DPHP (GM:0.764 ng/mL) in urine samples compared to other metabolites of OPFR. Consequently, it is of great significance to explore the role of DPHP in lipid metabolism. The impact of OPFR exposure on serum lipid profiles among the general population has remained largely uninvestigated. To our knowledge, this is the first research indicating that higher urinary DPHP concentrations were associated with lower serum TG levels based on a large-sample cross-sectional study. A previous study has suggested that DPHP could disturb cholesterol metabolism, observing dysregulated genes associated with cholesterol metabolism in chicken embryonic hepatocytes upon DPHP exposure [41]. Notably, laboratory studies have also revealed a similar association between DPHP exposure and inhibited lipid metabolism. Ruby [42] demonstrated a significant reduction of the fatty acid catabolic process in mice with chronic exposure to DPHP. Multiomics analysis of liver tissue showed decreased expression of genes involved in lipid catabolic processes and downregulation of the target gene of peroxisome proliferator-activated receptor gamma (PPARγ).

OPFRs constitute a class of emerging endocrine-disturbing chemicals, and the disrupted lipid metabolism may explain OPFR-induced obesity. Both in vitro and in vivo evidence have demonstrated that various types of OPFR can lead to lipid accumulation [24,43]. In particular, the activation of PPARγ and subsequent adipocyte differentiation have been implicated in the dysregulation of fat metabolism resulting from exposure to certain OPFRs [44,45]. Additionally, oxidative stress and lipid peroxidation have been shown to play a role in altered fat metabolism [46]. Studies have revealed that exposure to tris (2-chloroethyl) phosphate induces lipid accumulation in livers through interaction with nuclear reporter farnesoid X receptor and resulted in its downregulation [47]. The altered activity of the pregnane X receptor (PXR) and androgen receptor may also be involved in the metabolic disruption caused by OPFR exposure [48]. Research by Xiang suggested that PXR-mediated increased synthesis of fatty acid and suppressed β-oxidation played a role in disrupting lipid homeostasis induced by TCP [22]. Disorders in the biosynthesis of unsaturated fatty acids and steroid hormones, as well as alterations in the activity of the cytochrome P450 enzyme subfamily, contributed to lipid metabolism disorders [49]. Further research is needed to elucidate the underlying mechanisms of BCEP- and BDCPP-induced obesity.

The present research has several strengths. We investigated the impact of five OPFR metabolites on both obesity and serum lipid profiles in a large, representative, non-institutional population. Various confounding factors were considered and included in the fully adjusted regression models. Additionally, stratification analyses were performed to evaluate the role of OPFR exposure in different subgroups. However, there also exist potential limitations. Firstly, the cross-sectional study design limits our ability to establish causal relationships. Secondly, the assessment of OPFR exposure may be inaccurate because only spot urine samples were used to measure the concentrations of OPFR metabolites. Thirdly, we only explored five types of OPFR metabolites in the present study due to the limited data availability. Fourthly, a significant number of participants were excluded due to missing indispensable data for the present research, introducing potential source bias and limiting the generalizability of the research findings. Additionally, confounding factors may impact the results.

## 5. Conclusions

In summary, the present study suggests that OPFR exposure might increase the risk of developing obesity and dysregulated serum lipid levels. Future prospective research is needed to clarify the causal relationship and further explore the underlying mechanisms.

## Figures and Tables

**Table 1:** Sociodemographic, hematological, and health status characteristics of included participants in NHANES 2017–2018.

Variables	All Participants (*n* = 1334)
Age, years, mean ± SD	45.19 ± 17.59
Sex, *n* (%)	
Male	656 (49.18)
Female	678 (50.82)
BMI, kg/m^2^, mean ± SD	29.42 ± 7.88
Race, *n* (%)	
Mexican American	206 (15.44%)
Other Hispanic	122 (9.15%)
Non-Hispanic White	449 (33.66%)
Non-Hispanic Black	297 (22.26%)
Other race: including multiracial	260 (19.49%)
Smoking status, *n* (%)	
Current smoker	839 (62.89%)
Former smoker	206 (15.44%)
Non-smoker	289 (21.66%)
Drinking, *n* (%)	158 (11.84%)
Physical activity, *n* (%)	
None	675 (50.60%)
Moderate activity	299 (22.41%)
Vigorous activity	360 (26.99%)
Education level, *n* (%)	
Less than 9th grade	103 (8.25%)
9–11th grade	146 (11.70%)
High school graduate/GED or equivalent	281 (22.52%)
Some college or AA degree	406 (32.53%)
College graduate or above	312 (25.00%)
Marital status, *n* (%)	
Married/living with partner	747 (59.76%)
Widowed/divorced/separated	241 (19.28%)
Never married	262 (20.96%)
Family PIR, *n* (%)	
<1	248 (18.59%)
1–1.99	322 (24.14%)
2–3.99	296 (22.19%)
≥4	289 (21.66%)
Missing	179 (13.42%)
Hypertension, *n* (%)	334 (25.04%)
Diabetes, *n* (%)	105 (7.87%)
Stroke, *n* (%)	32 (2.40%)
Coronary artery disease, *n* (%)	20 (1.50%)
Heart failure, *n* (%)	20 (1.50%)
Heart attack, *n* (%)	237 (13.41%)

Abbreviations: BMI: body mass index; PIR: poverty income ratio.

**Table 2:** Urinary creatinine and metabolites of OPFR of included participants in NHANES 2017–2018.

Variables	All Participants (*n* = 1334)
Urinary creatinine (mg/dL), median (Q1–Q3)	118.00 (63.00–181.00)
Urinary metabolites of OPFRs (ng/mL), GM (95% CI)	
BCIPP	0.335 (0.313–0.357)
BCEP	1.046 (0.979–1.118)
BDCPP	0.136 (0.129–0.143)
DBUP	0.132 (0.125–0.138)
DPHP	0.764 (0.718–0.812)

**Table 3:** Associations between urinary OPFR metabolite concentrations and BMI.

Variables	Model 1		Model 2	
	*β* (95% CI)	*p* Value	*β* (95% CI)	*p* Value
BCIPP	0.32 (0.03, 0.62)	0.0323	0.17 (−0.15, 0.49)	0.3030
BCEP	0.33 (0.09, 0.57)	0.0068	0.27 (0.02, 0.52)	0.0338
BDCPP	0.71 (0.46, 0.96)	<0.0001	0.56 (0.25, 0.87)	0.0004
DBUP	0.35 (0.03, 0.67)	0.0310	0.07 (−0.28, 0.41)	0.6987
DPHP	0.26 (−0.00, 0.52)	0.0528	−0.08 (−0.40, 0.24)	0.6195

Abbreviations: CI: confidence interval. Model 1: adjusted for age, sex, and race. Model 2: adjusted for age, sex, race, smoking status, drinking status, physical activity, education level, marital status, family PIR, and urinary creatinine.

**Table 4:** Associations between urinary OPFR metabolite concentrations and obesity.

Variables	Models	OR (95% CI)	*p* Value
BCIPP	Model 1	1.05 (0.97, 1.13)	0.2557
	Model 2	1.02 (0.93, 1.11)	0.7364
BCEP	Model 1	1.12 (1.05, 1.20)	0.0005
	Model 2	1.10 (1.02, 1.18)	0.0096
BDCPP	Model 1	1.21 (1.13, 1.30)	<0.0001
	Model 2	1.19 (1.09, 1.30)	0.0001
DBUP	Model 1	1.10 (1.01, 1.20)	0.0273
	Model 2	1.04 (0.95, 1.14)	0.4258
DPHP	Model 1	1.09 (1.01, 1.17)	0.0206
	Model 2	1.00 (0.92, 1.09)	0.9820

Abbreviations: OR: odds ratio; CI: confidence interval. Model 1: adjusted for age, sex, and race. Model 2: adjusted for age, sex, race, smoking status, drinking status, physical activity, education level, marital status, family PIR, and urinary creatinine.

**Table 5:** Associations between urinary OPFR metabolite concentrations and lipid profiles.

Variables	Models	TG		TC		HDL-c		LDL-c	
		*β* (95% CI)	*p* Value	*β* (95% CI)	*p* Value	*β* (95% CI)	*p* Value	*β* (95% CI)	*p* Value
BCIPP	Model 1	−2.74 (−6.65, 1.18)	0.1714	−0.08 (−1.59, 1.42)	0.9125	−0.09 (−0.66, 0.48)	0.7540	−0.70 (−2.55, 1.16)	0.4628
	Model 2	−3.31 (−7.75, 1.13)	0.1444	0.05 (−1.63, 1.73)	0.9519	0.24 (−0.36, 0.84)	0.4345	−1.22 (−3.31, 0.86)	0.2499
BCEP	Model 1	0.61 (−2.62, 3.84)	0.7117	−0.51 (−1.71, 0.70)	0.4090	−0.54 (−1.00, −0.09)	0.0199	−0.41 (−1.94, 1.12)	0.6018
	Model 2	0.16 (−3.43, 3.75)	0.9305	−0.39 (−1.71, 0.92)	0.5580	−0.32 (−0.79, 0.16)	0.1887	−1.20 (−3.37, 0.97)	0.2794
BDCPP	Model 1	−0.88 (−4.45, 2.70)	0.6304	−0.78 (−2.06, 0.50)	0.2309	−0.55 (−1.04, −0.07)	0.0250	−0.41 (−2.11, 1.28)	0.6315
	Model 2	−2.25 (−6.88, 2.38)	0.3418	−0.82 (−2.46, 0.82)	0.3279	0.29 (−0.30, 0.89)	0.3299	−1.20 (−3.37, 0.97)	0.2794
DBUP	Model 1	1.04 (−3.56, 5.64)	0.6579	−0.45 (−2.05, 1.15)	0.5831	−0.40 (−1.01, 0.21)	0.1998	−0.31 (−2.49, 1.86)	0.7787
	Model 2	0.69 (−4.55, 5.93)	0.7961	−0.76 (−2.55, 1.03)	0.4055	0.10 (−0.54, 0.75)	0.7587	−1.30 (−3.75, 1.15)	0.2994
DPHP	Model 1	−5.67 (−9.34, −2.00)	0.0025	−0.67 (−1.99, 0.66)	0.3221	−0.59 (−1.09, −0.08)	0.0226	−1.03 (−2.78, 0.73)	0.2527
	Model 2	−7.41 (−12.13, −2.68)	0.0022	−0.17 (−1.83, 1.49)	0.8420	−0.06 (−0.65, 0.54)	0.8545	−1.20 (−3.44, 1.03)	0.2925

Abbreviations: TG: triglyceride; TC: total cholesterol; HDL-c: high-density-lipoprotein cholesterol; LDL-c: low-density-lipoprotein cholesterol; Model 1: adjusted for age, sex, and race. Model 2: adjusted for age, sex, race, BMI, smoking status, drinking status, physical activity, education level, marital status, family PIR, and urinary creatinine.

## Data Availability

Publicly available datasets were analyzed in this study. These data can be found at: https://www.cdc.gov/nchs/nhanes/index.htm (accessed on 1 March 2022).

## Supplementary Materials

The following supporting information can be downloaded at: https://www.mdpi.com/article/10.3390/metabo14020124/s1, Figure S1: Association between log2-transformed BCEP (A) and BDCPP (B) concentrations and odds ratio for obesity; Figure S2: Association between log2-transformed BCEP (A) and BDCPP (B) and odds ratio for obesity stratified by age; Figure S3: Association between log2-transformed BCEP (A) and BDCPP (B) and odds ratio for obesity stratified by sex; Figure S4: Association between log2-transformed BCEP (A) and BDCPP (B) and odds ratio for obesity stratified by race; Figure S5: Association between log2-transformed BCEP (A) and BDCPP (B) and odds ratio for obesity stratified by smoking status; Figure S6: Association between log2-transformed BCEP (A) and BDCPP (B) and odds ratio for obesity stratified by drinking status; Figure S7: Association between log2-transformed BCEP (A) and BDCPP (B) and odds ratio for obesity stratified by physical activity; Figure S8: Association between log2-transformed BCEP (A) and BDCPP (B) and odds ratio for obesity stratified by educational level; Figure S9: Association between log2-transformed BCEP (A) and BDCPP (B) and odds ratio for obesity stratified by marital status; Figure S10: Association between log2-transformed BCEP (A) and BDCPP (B) and odds ratio for obesity stratified by family PIR; Table S1: BMI value of participants across different OPFR metabolites concentrations; Table S2: Serum lipid profiles of participants across different OPFR metabolites concentrations.
