# Supplementary material for: Exposure to OPFRs Is Associated with Obesity and Dysregulated Serum Lipid Profiles: Data from 2017–2018 NHANES
Source: Metabolites. 2024 Feb 13;14(2):124. doi: 10.3390/metabo14020124 (PMC10890692; doi:10.3390/metabo14020124)
Supplement: Supplementary file 1 [file metabolites-14-00124-s001.zip › metabolites-2812729-Supplementary Figures.pdf]

Supplementary Figures

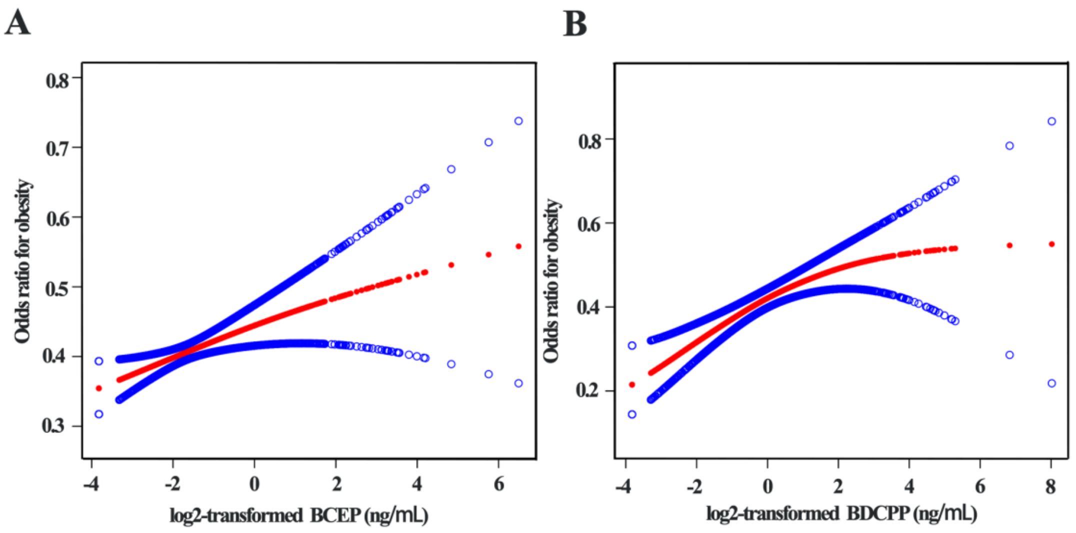

Figure S1. Association between log2-transformed BCEP (A) and BDCPP (B) concentrations and odds ratio for obesity.

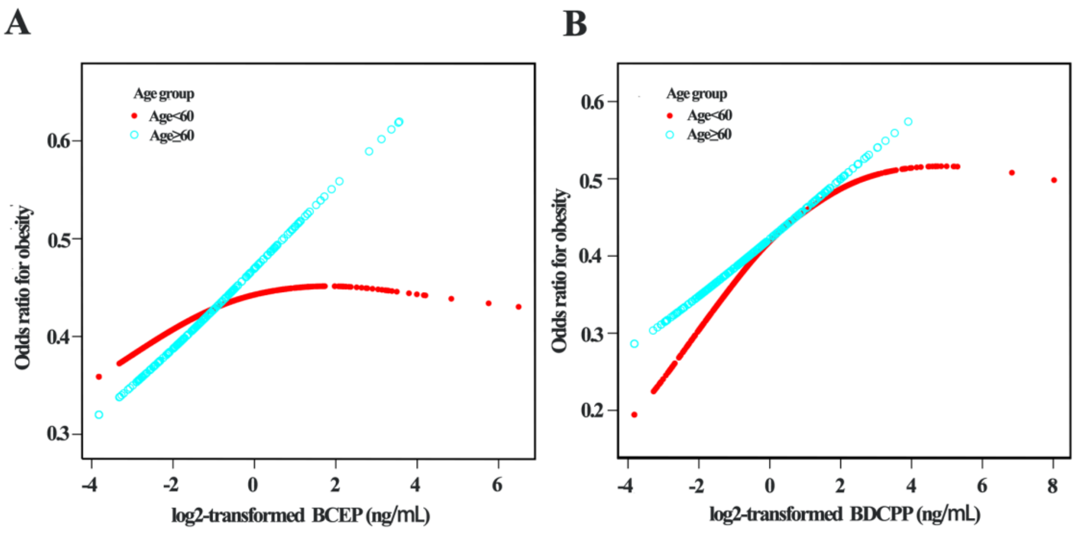

Figure S2. Association between log2-transformed BCEP (A) and BDCPP (B) and odds ratio for obesity stratified by age.

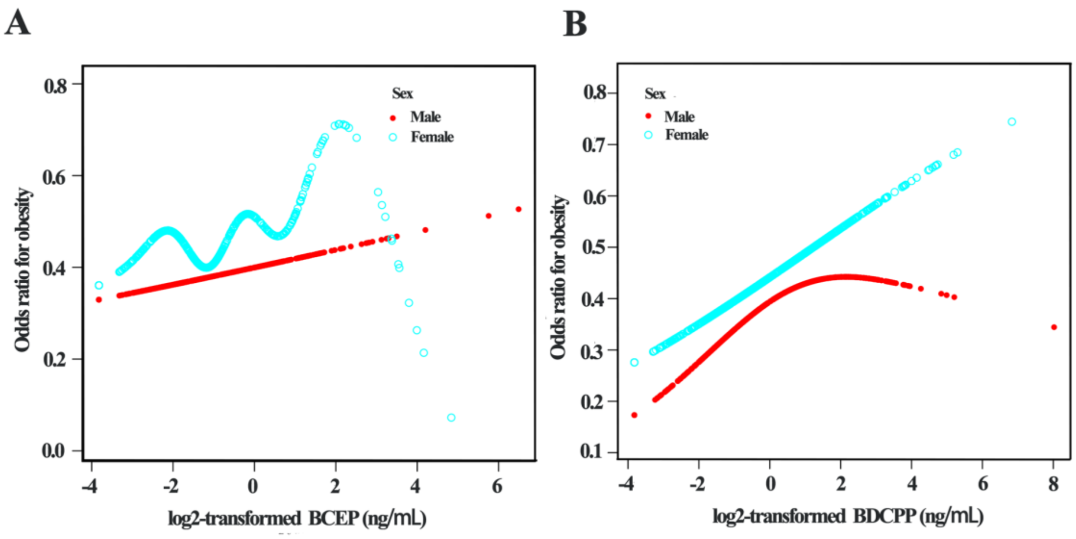

Figure S3. Association between log2-transformed BCEP (A) and BDCPP (B) and odds ratio for obesity stratified by sex.

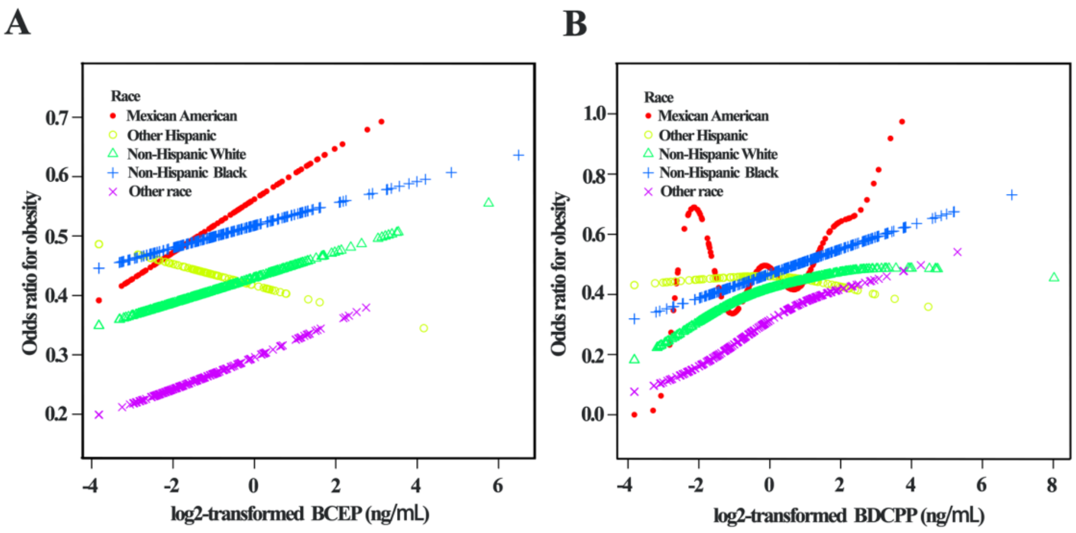

Figure S4. Association between log2-transformed BCEP (A) and BDCPP (B) and odds ratio for obesity stratified by race.

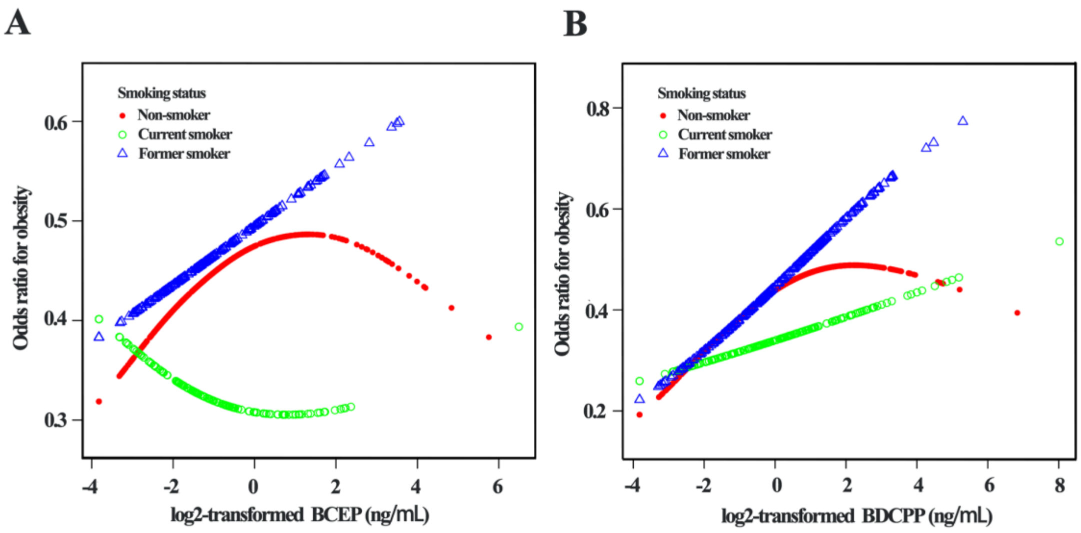

Figure S5. Association between log2-transformed BCEP (A) and BDCPP (B) and odds ratio for obesity stratified by smoking status.

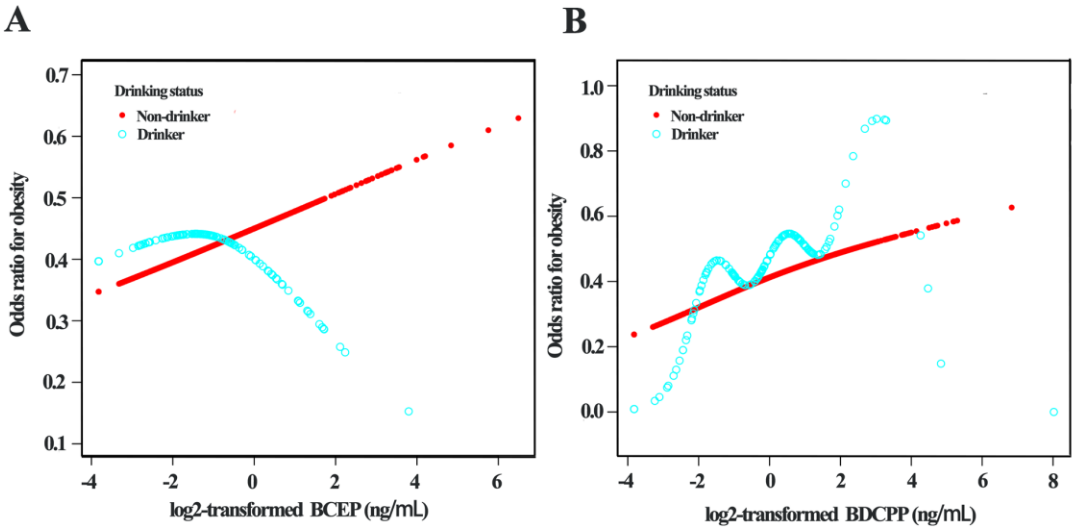

Figure S6. Association between log2-transformed BCEP (A) and BDCPP (B) and odds ratio for obesity stratified by drinking status.

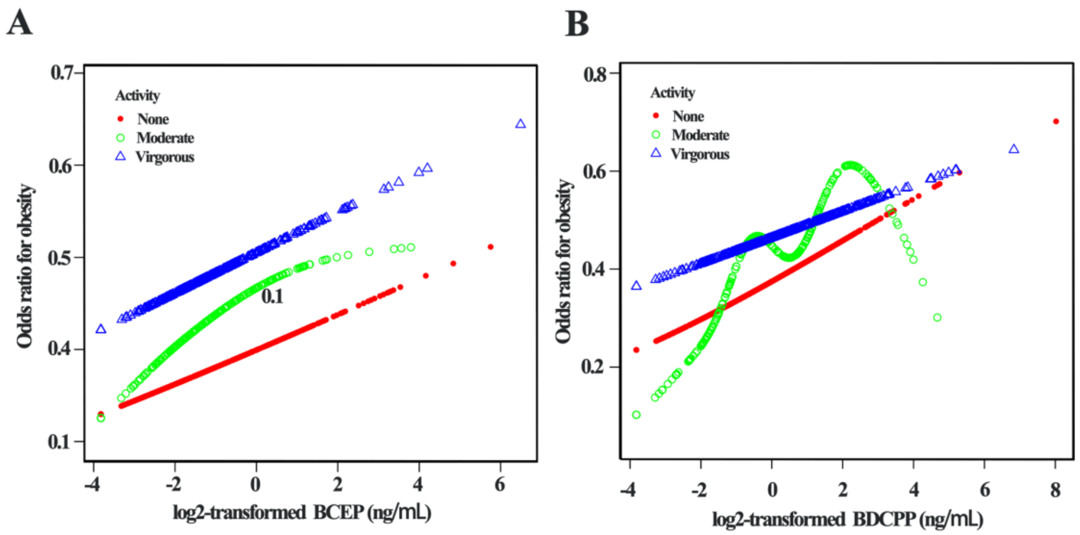

Figure S7. Association between log2-transformed BCEP (A) and BDCPP (B) and odds ratio for obesity stratified by physical activity.

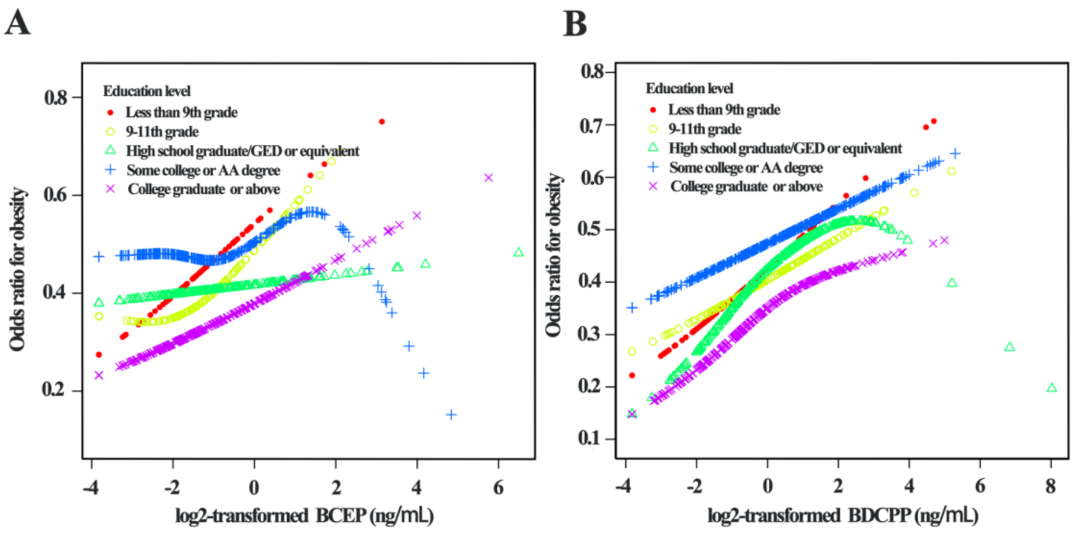

Figure S8. Association between log2-transformed BCEP (A) and BDCPP (B) and odds ratio for obesity stratified by educational level.

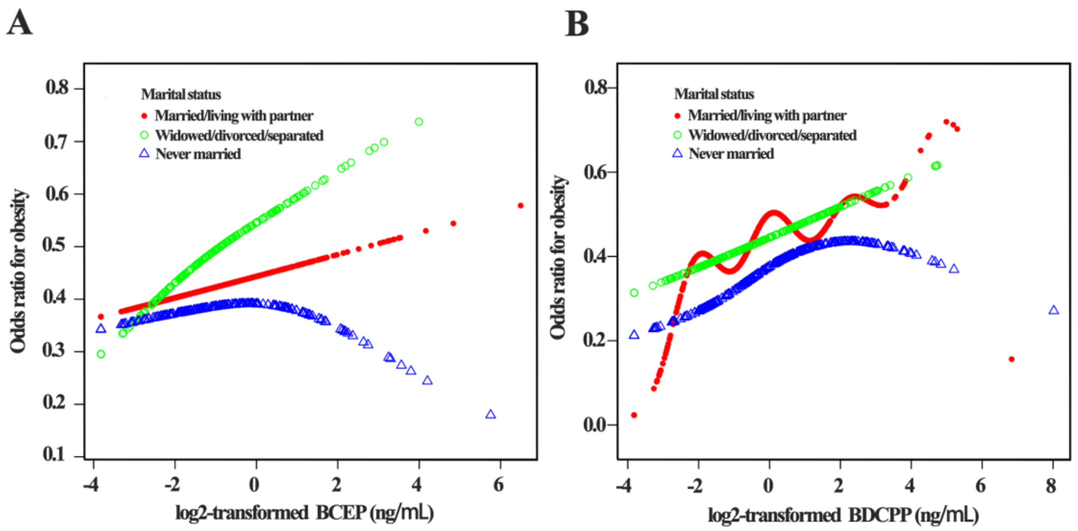

Figure S9. Association between log2-transformed BCEP (A) and BDCPP (B) and odds ratio for obesity stratified by marital status.

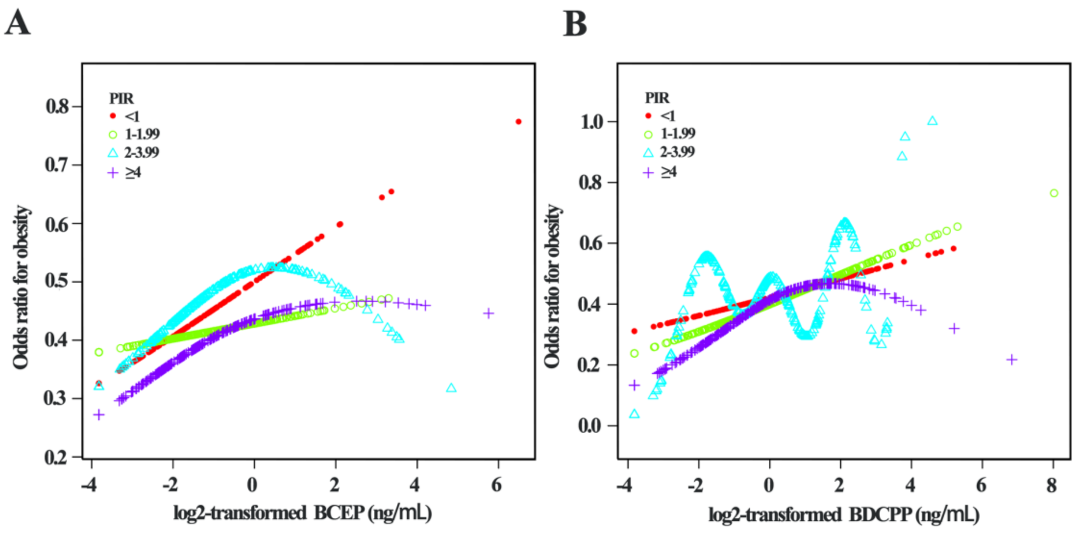

Figure S10. Association between log2-transformed BCEP (A) and BDCPP (B) and odds ratio for obesity stratified by family PIR.
