# Supplementary material for: Exposure to OPFRs Is Associated with Obesity and Dysregulated Serum Lipid Profiles: Data from 2017–2018 NHANES
Source: Metabolites. 2024 Feb 13;14(2):124. doi: 10.3390/metabo14020124 (PMC10890692; doi:10.3390/metabo14020124)
Supplement: Supplementary file 1 [file metabolites-14-00124-s001.zip › metabolites-2812729-Supplementary Tables.pdf]

Table S1. BMI value of participants across different OPFR metabolites concentrations

Variables	Q1	Q2	Q3	Q4	p value
BCIPP	30.05±9.48	30.12±7.90	30.60±8.37	29.79±7.72	0.283
BCEP	29.11±6.50	29.09±8.58	30.67±9.35	30.18±8.16	0.211
BDCPP	27.63±6.33	28.88±7.64	30.00±7.45	31.64±9.54	<0.001
DBUP	29.37±7.55	29.84±7.02	31.93±10.22	30.07±8.09	0.209
DPHP	28.41±7.81	29.93±8.34	30.39±8.24	29.74±7.77	0.118

Table S2. Serum lipid profiles of participants across different OPFR metabolites concentrations

Variables	Q1	Q2	Q3	Q4	P value
TG					
BCIPP	1.08±0.59	1.14±0.64	1.10±0.66	1.01±0.59	0.498
BCEP	1.15±0.64	1.11±0.70	1.08±0.65	1.12±0.62	0.537
BDCPP	1.14±0.71	1.20±0.72	1.08±0.55	1.01±0.63	0.021
DBUP	1.21±0.70	1.14±0.65	0.95±0.55	1.15±0.69	0.061
DPHP	1.26±0.74	1.10±0.56	1.09±1.65	0.97±0.59	<0.001
TC					
BCIPP	4.87±0.63	4.63±0.99	4.71±1.05	4.80±0.83	0.692
BCEP	4.78±1.01	4.87±1.05	4.82±1.02	4.73±0.97	0.712
BDCPP	5.01±1.15	4.97±1.1.3	4.78±0.98	4.63±0.88	<0.001
DBUP	4.86±0.84	4.77±0.98	4.49±1.03	4.90±1.07	0.602
DPHP	5.13±1.14	4.86±1.07	4.17±0.95	4.66±0.95	<0.001
HDL-c					
BCIPP	1.37±0.30	1.30±0.32	1.31±0.38	1.44±0.47	0.322
BCEP	1.36±0.41	1.43±0.46	1.37±0.35	1.36±0.45	0.207
BDCPP	1.41±0.41	1.43±0.44	1.35±0.32	1.36±0.38	0.067
DBUP	1.35±0.43	1.35±0.39	1.37±0.39	1.44±0.52	0.853
DPHP	1.45±0.44	1.35±0.38	1.36±0.36	1.41±0.48	0.492
LDL-c					
BCIPP	3.01±0.94	2.81±0.85	2.90±0.95	2.89±0.78	0.710
BCEP	2.89±0.88	2.93±0.91	2.96±0.91	2.86±0.86	0.891
BDCPP	3.05±1.03	3.00±0.99	2.93±0.84	2.80±0.80	0.227
DBUP	2.96±0.92	2.90±0.96	2.68±0.96	2.93±0.78	0.094
DPHP	3.10±1.01	3.01±1.01	2.84±0.84	2.81±0.77	0.028
